# The effects of creatine and glycerol hyperhydration on running economy in well trained endurance runners

**DOI:** 10.1186/1550-2783-8-24

**Published:** 2011-12-16

**Authors:** Lukas Y Beis, Thelma Polyviou, Dalia Malkova, Yannis P Pitsiladis

**Affiliations:** 1 College of Medicine, Veterinary and Life Sciences, Institute of Cardiovascular and Medical Sciences, University of Glasgow, Glasgow, UK; 2 Department of Human Nutrition, Queen Mother Hospital, Tower Block, Glasgow, G3 8SJ, UK

## Abstract

**Background:**

Ingestion of creatine (Cr) and glycerol (Gly) has been reported to be an effective method in expanding water compartments within the human body, attenuating the rise in heart rate (HR) and core temperature (T_core_) during exercise in the heat. Despite these positive effects, a substantial water retention could potentially impair endurance performance through increasing body mass (BM) and consequently impacting negatively on running economy (RE). The objective of the present study was to investigate the effects of a combined Cr and Gly supplementation on thermoregulatory and cardiovascular responses and RE during running for 30 min at speed corresponding to 60% of maximal oxygen uptake $\left(\dot{\text{V}}{\text{O}}_{\text{2}}\max\right)$ in hot and cool conditions.

**Methods:**

Cr·H_2_O (11.4 g), Gly (1 g·kg^-1 ^BM) and Glucose polymer (75 g) were administered twice daily to 15 male endurance runners during a 7-day period. Exercise trials were conducted pre- and post-supplementation at 10 and 35°C and 70% relative humidity.

**Results:**

BM and total body water increased by 0.90 ± 0.40 kg (*P *< 0.01; mean ± SD) and 0.71 ± 0.42 L (*P *< 0.01), respectively following supplementation. Despite the significant increase in BM, supplementation had no effect on $\dot{\text{V}}{\text{O}}_{\text{2}}$ and therefore RE. Both HR and T_core _were attenuated significantly after supplementation (*P *< 0.05, for both). Nevertheless, thermal comfort and rating of perceived exertion was not significantly different between pre- and post-supplementation. Similarly, no significant differences were found in sweat loss, serum osmolality, blood lactate and in plasma volume changes between pre- and post-supplementation.

**Conclusions:**

Combining Cr and Gly is effective in reducing thermal and cardiovascular strain during exercise in the heat without negatively impacting on RE.

## Background

Running economy (RE), which is defined as the sub-maximal oxygen consumption $\left(\dot{\text{V}}{\text{O}}_{\text{2}}\right)$ at a given running velocity, is an important physiological parameter as superior RE is essential for successful endurance running performance [1,2]. In general, runners with good RE use less oxygen than runners with poor RE at the same absolute exercise intensity. RE appears to be influenced by many physiological factors [1] including hydration status. Coyle (2003) proposed that a -4 to -8% body mass (BM) deficit due to dehydration (i.e., the process of reducing body water) may lower the oxygen cost of movement [3], given that athletes who lose the most BM during a race are usually the most successful [4]. Nevertheless, this theoretical paradigm contradicts the prevailing view of a body water deficit in excess of 2-3% BM constituting the level of dehydration that can adversely affect performance [5].

During exercise, skeletal muscle produces a significant amount of heat. When this metabolic heat production exceeds total heat loss, core body temperature (T_core_) rises. Consequently, endurance exercise performance in hot and dry environments can be limited by the increase in T_core _[6]. An increase in T_core _during can be attenuated via the secretion and evaporation of sweat through the skin with inevitable body water loss. This decrease in body water is hypothesized to decrease plasma volume (PV) and consequently reduce the sweating response and therefore thermoregulation capacity, increase heart rate (HR) and reduce skin blood flow [7]. Improved maintenance of PV is the overriding rationale for fluid ingestion during exercise by those supportive of the "cardiovascular model of dehydration" [5]. However, proposed guidelines [5] are not always practical (e.g., difficulties providing adequate drinks during a race, athletes difficulties in drinking while running) and athletes typically refrain from consuming recommended amounts of fluids. Other means to expand PV can be by infusion of isotonic saline [8] with somewhat conflicting success [8,9]. More recent approaches aimed at expanding body water compartments using hydrating agents such as creatine (Cr) and glycerol (Gly) have successfully attenuated the rise in T_core _and HR during exercise in heat [10,11].

Cr has been shown to have hydrating effects [12,13], although the exact process has yet to be established. Ingestion of 20 g·d^-1 ^of Cr dissolved in 500 mL of water for 7 days have proved successful in attenuating the rise in HR and T_core _during exercise in the heat [13]. These effects have been attributed to an increase in intracellular water (ICW), resulting in an increased specific heat capacity of the body [12,13]. Moreover, whole body Cr retention is 60% higher when consumed with carbohydrate (CHO) compared to when Cr was consumed alone [14]. Although the mechanism by which CHO enhances Cr uptake is not completely understood, consumption of 100 g per 5 g of Cr has been recommended for the effective improvement of Cr uptake [15]. Like Cr, Gly has been found to be an effective agent in expanding the water compartments within the human body [11,16]. Gly, seems to expand the ICW as well as the extracellular water (ECW) [17]. In general, doses of 1.0-1.5 g Gly·kg^-1 ^BM dissolved in 1.4 - 2.0 L of fluid 2.5 - 4 h before exercise [18] increase total body water (TBW) compartments and reduce thermal and cardiovascular strain during exercise in the heat.

Supplementation with combined hydrating agents such as Gly or Cr has consistently produced modest fluid retention of 400 - 800 mL [10-12]. Easton et al. (2007) were the first to add Gly to a Cr containing solution and demonstrate that a combination of the two hyperhydrating agents has an additive effect, as the addition of Gly to Cr significantly increased TBW more than Cr alone. Although the combination of the aforementioned hyperhydrating agents results in an increase in TBW and a reduction in certain cardiovascular and thermoregulatory responses [19], the BM increase due to enhanced hydration status could potentially reduce RE. The reduction of the energy cost of movement at a sub-maximal velocity by way of reducing BM to improve running performance is well known [20]. For instance, it is noted that some marathon runners perform well despite dehydration of 4-8% BM [21]. Coyle [3] proposed that this may occur because fluid loss (i.e., reduced body mass) lowers the oxygen cost of movement. On the other hand, the acute influences of hyperhydration on RE has not been investigation to date. Hence, the aim of the present study was to investigate the effects of hyperhydration induced by a combined Cr and Gly supplementation on thermoregulatory and cardiovascular responses and RE during 30 min of running at a running speed corresponding to 60% $\dot{\text{V}}{\text{O}}_{\text{2}}\max$ in cool (10°C with a relative humidity of 70%) and hot conditions (35°C with a relative humidity of 70%) in well trained male athletes. In cool ambient conditions were intended to minimize heat stress during exercise this enabling a focus on the effects of the altered BM induced by hyperhydration on RE at 60% $\dot{\text{V}}{\text{O}}_{\text{2}}\max$. However, effects of hyperhydration on thermoregulatory and cardiovascular responses are also expected during exercise in hot and humid conditions; conditions typical of major sporting events (e.g., Olympic Summer Games). As such, it was hypothesized that an increase in BM and TBW induced by hydrating agents such as Gly or Cr would improve thermoregulatory and cardiovascular responses in line with previous findings but potentially negatively influence RE during running in the heat.

## Methods

### Subjects

Fifteen trained male runners gave their written informed consent to take part in the present study which was approved by the University of Glasgow Ethics Committee and was performed according to the code of ethics of the World Medical Association (Declaration of Helsinki). One subject withdrew from the study before the final trial because of gastrointestinal distress during supplementation. Subjects were questioned as to their supplementation and training practices in order to ascertain that they had not supplemented with Cr for at least 8 weeks prior to commencing the study. Subjects were in good health at the time of testing, ran on a daily basis and participated regularly in competitive races. Athletes were also requested to maintain their typical weekly training regime during the course of the study.

### Study design: Preliminary exercise tests

All subjects completed a $\dot{\text{V}}{\text{O}}_{\text{2}}\max$ test during an initial continuous incremental test at standard room temperature (20 - 21°C) and relative humidity (30 - 40%) on a motorized treadmill (PPS Med, Woodway, Germany) at 1% grade. After a warm-up period (depending on the runner), the subjects started running at 8 km·h^-1 ^for 3 min in order to reach a steady state. In the next exercise bout the treadmill speed was set to 10 km·h^-1 ^for 3 min and this procedure was repeated with 2 km·h^-1 ^increments in running speed until volitional exhaustion of the subject. During the test expired gas samples (30 s collection time at the end of each bout) were taken using Douglas bag collection technique as is considered the gold standard method [22] and analyzed for O_2_% and CO_2_% (Servopro 4100 Gas Purity Analyzer, Servomex, UK) as well as analyzed for volume using a dry gas meter (Harvard, Kent, UK) and temperature of expired gases. Barometric pressure was measured using a standard mercury barometer. Additionally, a HR monitor (Polar Sports Tester, Polar Electro Oy, Kempele, Finland) was attached prior to each test and HR was recorded at the end of each bout. The $\dot{\text{V}}{\text{O}}_{\text{2}}\max$ measurement was used for calculating the intensity (60% of $\dot{\text{V}}{\text{O}}_{\text{2}}\max$) that subjects would perform during the actual tests. Running speed at 60% of $\dot{\text{V}}{\text{O}}_{\text{2}}\max$ (exercise intensity) was calculated using the linear relation between treadmill speed and $\dot{\text{V}}{\text{O}}_{\text{2}}$.

**Figure 1:**
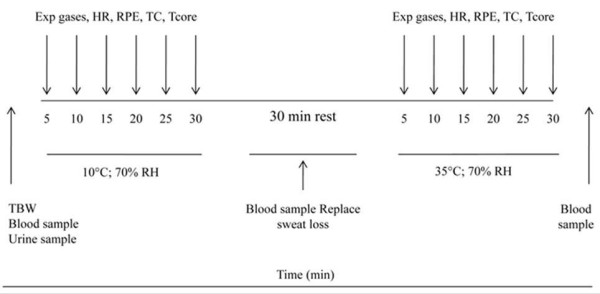
**Schematic representation of the experimental protocol**.

Prior to the actual experimental trials, familiarization trials were completed until the variability of $\dot{\text{V}}{\text{O}}_{\text{2}}$ of two consecutive trials was within 5% difference. No subject had to complete a third familiarization trial to achieve less than 5% variability, an observation which is in line with our previous experience of trained runners [23]. At least three days after this familiarization period, subjects reported to the laboratory for the first experimental trial (i.e., a pre-supplementation trial). After this baseline test, all subjects commenced the hyperhydration treatment comprising Cr, Gly and Glu. For this, subjects consumed a solution of 11.4 g of Cr·H_2_O (equivalent to 10 g Cr), (Reflex Creapure Creatine, Reflex Nutrition LTD, UK), 1 g·kg^-1 ^of BM Gly (Glycerin BP/Value Health Glycerin BP, Boots Company plc) and 75 g of Glu polymer (SiS GO electrolyte), mixed in hot water (approximately 50°C) and made up in 1 L of cold water twice daily. This supplementation regimen was followed for 6 days. This protocol has been shown to increase resting muscle-phosphocreatine levels within 5 days [24]. On the day of the post-supplementation test (i.e., day 7^th^) subjects began consuming the final supplement 5 h before the exercise-performance trial (with instructions to complete ingestion within 1 h). Hypertonic solutions such as the Cr, Gly, Glu combination (~1556 mOsm·kg^-1^) cause an initial net secretion of water into the intestinal lumen [25], resulting in an effective loss of body water, albeit temporary. Unpublished work from our laboratory has indicated that ingesting Cr/Gly 5 h prior to commencement of exercise results in a larger volume of fluid absorbed compared to when the solution is consumed 3 h prior to the exercise test. In order to prevent degradation of Cr to creatinine, each supplement was prepared fresh each time before consumption. The subject was also given a temperature pill (HQ Inc., USA) about 8-12 h prior to each test allowing T_core _to be measured [26]. On each of the experimental test days, subjects ingested 500 mL of water 1 h before exercise in an attempt to ensure euhydration before all exercise trials [27] (Figure 1). Subjects otherwise followed their normal diet and recorded all food and drink consumed during the supplementation period as well as the preceding week using a food diary. The diet was analyzed for energy intake and macronutrient content using computerized food-composition tables [28] (Food Meter U.K., Medimatica s.r.l., Benedetto, Italy). Subjects were asked to minimize caffeine intake to 1 cup of tea or coffee per day to lessen any possible confounding effects of caffeine on Cr [29].

### Experimental Procedures

**Figure 2:**
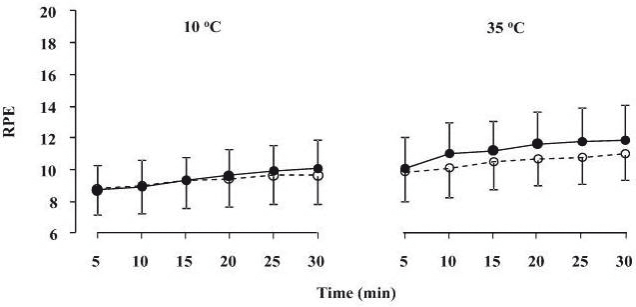
**Rating of perceived exertion (RPE) during exercise at 10 and 35°C before (black circles) and after (white circles) supplementation**. Data presented as mean ± SD.

**Figure 3:**
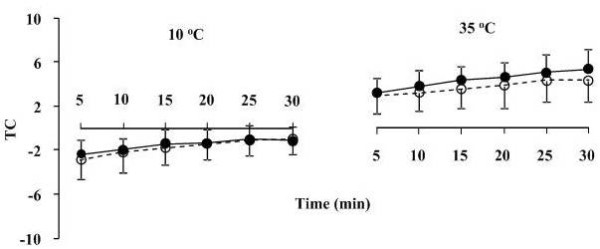
**Thermal comfort (TC) during exercise at 10 and 35°C before (black circles) and after (white circles) supplementation**. Data presented as mean ± SD.

The subject reported to the lab after a 3 h fast and having refrained from alcohol, caffeine, and strenuous exercise at least 24 h prior to the experimental trial. Firstly, a urine sample was collected from the subject prior to taking the pre-test nude BM (Tanita Corporation of America, Inc.). Body water compartments were estimated using a multi frequency bioimpedance analyzer (Quadscan 4000, Bodystat Ltd., Isle of Man) while the subject lay comfortably in a supine position for 5 min on a nonconductive surface with their arms and legs slightly abducted. This method allows TBW and ECW to be estimated. From these measurements ICW can also be deduced. Bioimpedance has been shown to produce valid and reliable TBW estimations in the euhydrated state [30]. To date, several studies have successfully used this technique in order to estimate hyperhydration induced changes in TBW [12,13]. Changes in BM from pre- to post-supplementation were used to supplement the indirect measurement of the fluid volume retained. Following TBW determination, the subject lay in a supine position for 5 min further and a 7 mL blood sample was taken from a 21G cannula which was introduced into a superficial vein of the anticubital fossa of the right arm. The venous cannula was kept patent by flushing it with 7 mL of isotonic saline solution between samples. Prior entering the environmental chamber a HR monitor (Polar Sports Tester, Polar Electro Oy, Kempele, Finland) was attached to the subject. Then, the subject was transferred to the climatic chamber (ambient temperature 10.0 ± 1.0°C with a relative humidity of 68.5 ± 3.6%. Subjects were then instructed to begin running to their predetermined 60% $\dot{\text{V}}{\text{O}}_{\text{2}}\max$ for 30 min at 1% inclination of the treadmill. HR and T_core _were recorded every 5 min throughout the 30-min exercise period. 1 min gas measurements were collected at 5 min intervals of exercise for the purpose of $\dot{\text{V}}{\text{O}}_{\text{2}}$, carbon-dioxide production $\left(\dot{\text{V}}\text{C}{\text{O}}_{\text{2}}\right)$, temperature and expired gas volume determination. Rating of perceived exertion (RPE; Figure 2) and thermal comfort (TC; Figure 3) were recorded every 5 min of the exercise using the Borg category scale [31] for RPE and a modified scale (from -10 to +10). Following the first exercise bout, the subject was removed from the chamber and nude BM was measured immediately. The difference in BM before and after exercise was calculated and subsequently used to estimate sweat loss. Subsequent to BM determination, the subject lay in a supine position for 10 min and a final blood sample was retrieved. The fluid loss was then replaced by giving the subject the equivalent amount of water to that calculated between pre- and post-exercise. Subjects were then instructed to re-enter the climatic chamber and complete a second bout of run at the same speed (60% $\dot{\text{V}}{\text{O}}_{\text{2}}\max$), at 35.1 ± 0.1°C and 69.4 ± 4.0% relative humidity. The protocol for data collection was identical to the one used in the first bout of exercise. Once the second bout was completed, subjects' nude BM and a final blood sample were taken as described above. The analytical procedure is shown in Figure 1.

Blood was drawn into dry syringes and 4 mL dispensed into a tube containing K_3_EDTA and the remaining 3 mL dispensed into plain tubes. Duplicate aliquots (100 μL) of whole blood from the K_3_EDTA tube were rapidly deproteinized in 1000 μL of ice-cold 0.3-mmol/L perchloric acid, centrifuged (8 min, 14000 rpm, HettichMicrocentrifuge, Germany), and frozen for later analysis of lactate using a standard enzymatic method [32] involving fluorimetric detection (Spectramax M2 Microplate Reader, Molecular Devices, Inc., US). The blood in tubes without anticoagulant was allowed to coagulate and then centrifuged; the serum collected was used to measure osmolality by freezing-point depression (Micro-osmometer 3300, Vitech Scientific, West Sussex, UK). The blood from the K_3_EDTA tubes was also analyzed for hemoglobin (cyanmethemoglobin method) and packed-cell volume (conventional microhematocrit method). All blood analyses were carried out in duplicate, with the exception of packed-cell volume, which was carried out in triplicate. PV changes were calculated from changes in hemoglobin and packed-cell volume relative to initial baseline values [33].

### Statistical analysis

All data are expressed as the mean ± SD. All experimental variables ($\dot{\text{V}}{\text{O}}_{\text{2}}$, $\dot{\text{V}}\text{C}{\text{O}}_{\text{2}}$, RER, RPE, TC, HR, T_core_) were tested for normality of distribution and compared between the two treatments using a repeated measures two-way analysis of variance (ANOVA) (i.e., pre- vs. post-supplementation). Students paired t-tests were carried out to test difference between each time pre- to post-supplementation when difference was detected using ANOVA. Statistical significance was set at *P *< 0.05 and in cases where significant differences were detected between time points pre- to post-supplementation, *P*-value was corrected using the Sidak adjustment. Responses at 10 and 35°C were analysed separately. Student paired t-tests were also used to examine the difference between pre- to post-supplementation for the rest of the comparisons. All statistical analysis was completed using the statistical package SPSS, version 15.0 (Statistica 8.0, Statsoft Inc., Tulsa, USA).

## Results

### Subject characteristics

The 15 male subjects were trained distance runners with $\dot{\text{V}}{\text{O}}_{\text{2}}\max$ being 63.5 ± 5.2 ml·kg^-1^·min^-1^, age, 24 ± 5 yr; height, 180 ± 7 cm; BM, 69.5 ± 5.0 kg (values are presented as the mean ± SD).

### Body Mass and Water Compartments

**Figure 4:**
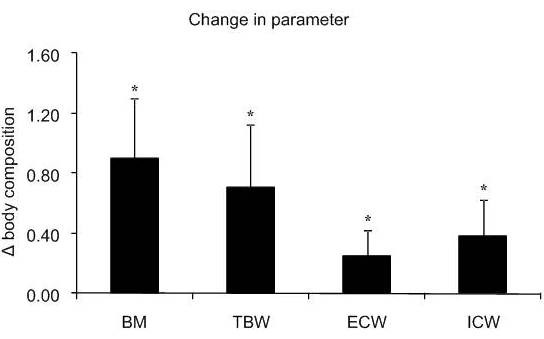
**Changes in body mass (BM), total body water (TBW), extracellular water (ECW) and intracellular water (ICW) induced by supplementation**. Data presented as mean ± SD. *Significant difference between pre- and post-supplementation. The units for Δ body composition are kg for BM and L for body water compartments.

Supplementation induced significant increase in BM, TBW, ICW and ECW (*P *< 0.01; Figure 4). During supplementation period as well as the preceding week averaged daily energy intake (Pre: 12.8 ± 2.1 MJ·d^-1^; Post: 11,5 ± 2.4 M J·d^-1^) and averaged proportion of energy obtained from carbohydrate (Pre: 55 ± 5%; Post: 49 ± 11%), fat (Pre: 33 ± 5% Post: 36 ± 6%), and protein (Pre: 13 ± 1%; Post: 14 ± 3%) were not significant different.

### Cardiopulmonary Variables

**Figure 5:**
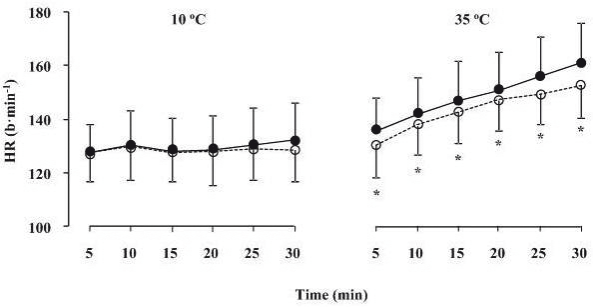
**Heart rate (HR) during exercise at 10 and 35°C before (black circles) and after (white circles) supplementation**. Data presented as mean ± SD. *Significant difference between pre- and post-supplementation.

Over the duration of running at 10°C $\dot{\text{V}}{\text{O}}_{\text{2}}$, $\dot{\text{V}}\text{C}{\text{O}}_{\text{2}}$ and respiratory exchange ratio (RER) remained constant (Table 1). Over the duration of running at 35°C $\dot{\text{V}}{\text{O}}_{\text{2}}$ and $\dot{\text{V}}\text{C}{\text{O}}_{\text{2}}$ increased significantly (*P *< 0.05, AVOVA, time effect) while the values of RER were constant. No significant differences were detected for $\dot{\text{V}}{\text{O}}_{\text{2}}$, $\dot{\text{V}}\text{C}{\text{O}}_{\text{2}}$, RER between pre- and post-supplementation trials during running at both 10 and 35°C (Table 1). HR increased significantly over the duration of running at 10 and 35°C (*P *< 0.05, for both, ANOVA, time effect). During running at 10°C there was no difference in HR between pre-and post-supplementation trials (Figure 5). During running at 35°C, HR was significantly lower (*P *< 0.05, ANOVA, trial effect) in the post-supplementation trial compared to the pre-supplementation trial.

**Table 1:** Oxygen consumption $\left(\dot{\text{V}}{\text{O}}_{\text{2}}\right)$, carbon dioxide production $\left(\dot{\text{V}}\text{C}{\text{O}}_{\text{2}}\right)$, respiratory exchange ratio (RPE) during 30 min of running at 10 and 35°C conducted before and after supplementation.

			Exercise time (min)					
**Variable**	**Condition**		**5**	**10**	**15**	**20**	**25**	**30**

$\dot{\text{V}}{\text{O}}_{\text{2}}$ (mL·kg^-1^·min^-1^)	10°C	Pre	37.4 ± 2.4	37.6 ± 2.0	37.7 ± 1.8	38.7 ± 2.2	38.8 ± 2.7	38.9 ± 2.8
		Post	36.4 ± 2.8	37.4 ± 1.5	36.9 ± 1.7	37.7 ± 1.8	37.6 ± 2.2	38.4 ± 3.3
	35°C	Pre^a^	37.2 ± 2.4	39.5 ± 2.4	39.5 ± 2.3	40.3 ± 2.6	40.5 ± 4.4	41.2 ± 3.3
		Post^a^	36.7 ± 2.4	37.9 ± 2.3	37.4 ± 3.2	38.4 ± 2.6	39.1 ± 2.1	38.5 ± 3.1
$\dot{\text{V}}\text{C}{\text{O}}_{\text{2}}$ (mL·kg^-1^·min^-1^)	10°C	Pre^a^	32.8 ± 1.7	33.7 ± 2.2	33.9 ± 1.4	34.4 ± 2.0	34.7 ± 2.5	34.4 ± 2.5
		Post^a^	33.5 ± 3.1	34.6 ± 1.6	34.0 ± 1.7	35.0 ± 1.9	35.1 ± 2.0	35.0 ± 2.3
	35°C	Pre	32.3 ± 2.8	34.7 ± 2.3	35.6 ± 2.3	35.3 ± 2.2	35.5 ± 3.2	35.5 ± 3.3
		Post	32.4 ± 2.5	33.9 ± 2.2	34.4 ± 2.4	35.1 ± 2.3	35.1 ± 2.3	34.5 ± 2.6
RER	10°C	Pre	0.87 ± 0.03	0.89 ± 0.03	0.89 ± 0.03	0.88 ± 0.04	0.89 ± 0.04	0.88 ± 0.03
		Post	0.91 ± 0.05	0.93 ± 0.03	0.92 ± 0.03	0.93 ± 0.03	0.93 ± 0.03	0.92 ± 0.03
	35°C	Pre	0.87 ± 0.05	0.88 ± 0.03	0.89 ± 0.03	0.88 ± 0.04	0.88 ± 0.05	0.86 ± 0.05
		Post	0.88 ± 0.03	0.89 ± 0.03	0.91 ± 0.03	0.91 ± 0.03	0.90 ± 0.03	0.89 ± 0.03

*Note. Values are presented as the mean ± SD. ^a^Significant difference over time throughout the trial. *P*-value was set at 0.05.

### Rating of Perceived Exertion (RPE) and Thermal Comfort (TC)

Over the duration of running conducted at both 10 and 35°C significant (*P *< 0.05, ANOVA, time effect) increases were detected in RPE (Figure 2) and TC (Figure 3), while no significant differences were found between pre- and post-supplementation trials.

### Core Temperature

**Figure 6:**
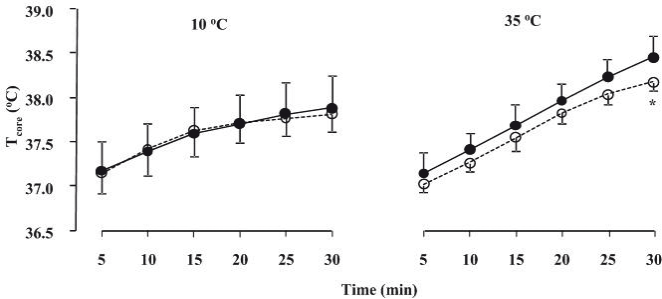
**Core temperature (T_core_) during exercise at 10 and 35°C before (black circles) and after (white circles) supplementation**. Data presented as mean ± SD. *Significant difference between pre- and post-supplementation.

Over the duration of running conducted at both 10 and 35°C T_core _increased significantly (*P *< 0.05, for both, ANOVA, time effect) (Figure 6). During running at 35°C T_core _was significantly lower (*P *< 0.01, ANOVA, trial effect) in post- than pre- supplementation trial. During running at 10°C there was no difference in T_core _between pre- and post-supplementation trials.

### Urine osmolality

No significant changes were found in urine osmolality between the pre- (438 ± 306 mOsm·kg^-1^) and post-supplementation trials (448 ± 266 mOsm·kg^-1^).

### Total Sweat Loss

During running at 10°C no significant differences between pre- and post-supplementation trials were observed in sweat loss (Pre: 0.3 ± 0.1 L; Post: 0.3 ± 0.1 L). Similarly, during running at 35°C no significant differences between pre- and post-supplementation trials were observed in sweat loss (Pre: 0.7 ± 0.2 L; Post: 0.8 ± 0.2 L).

### Blood Lactate and Plasma Volume

During running at both 10 and 35°C no significant differences were found between pre- and post-supplementation trials in resting concentration of blood lactate. Furthermore, no significant increase in blood lactate was observed over duration of exercise. Additionally, during running at both 10 and 35°C no significant differences were detected between pre- and post-supplementation trials in PV changes.

### Osmolality

Resting serum osmolality did not differ between pre- (268 ± 9 mOsm·kg^-1^) and post-supplementation (271 ± 19 mOsm·kg^-1^) trials. Additionally, no differences were observed between the post 10 and 35°C bouts and the resting values or between the treatments.

### Side Effects

In general, subjects tolerated the supplementation protocol well, with only 1 report of gastrointestinal distress after supplementation who withdrew from the experimental process before completing the post-supplementation trial. This report is in line with the previous study by Easton et al. (2007), where 1 athlete had to also withdraw from the study due to similar reasons.

## Discussion

The novel finding of this study is that a previously established pre-exercise water loading strategy using a combination of hydrating agents such as Cr and Gly that significantly increased body water compartments and reduced cardiovascular (Figure 5) and thermoregulatory (Figure 6) responses during running at 35°C, had no effect on the oxygen cost of running at 60% of $\dot{\text{V}}{\text{O}}_{\text{2}}\max$. The magnitude of change in BM following hyperhydration was similar to that previously reported in our laboratory [19] and by Kern et al. (2001). Somewhat smaller differences in body water compartments were observed in the present study compared to the previous investigation by Easton et al. (2007). For example, Easton et al [19] reported an increase of 0.9 L in TBW and 0.5 L in ICW after 7 days of supplementation. In the present study TBW and ICW were elevated by 0.7 and 0.3 L respectively after 7 days of supplementation. These differences could only be attributed to individual responses (i.e., level of "responders" to Cr supplementation as previously demonstrated) [13,34] as similar protocols were utilised. In the present study, the retained water was dispersed in both the ICW and ECW. Despite the significant increase in BM and body water compartments and consequently improved thermoregulatory responses during exercise, no significant differences in any of the respiratory variables were found between the pre- and post-supplementation exercise trials. Therefore, the finding that a significant increase in BM did not negatively impact on RE of trained runners supports the use of hyperhydration during endurance running when running in hot and humid conditions although confirmatory results are required during faster running speeds typical of sporting competition (i.e., > 85% $\dot{\text{V}}{\text{O}}_{\text{2}}\max$).

Temperature and cardiovascular regulation during exercise in the heat do appear to be critically dependent on hydration status [35,36]. In the present study, combined Cr and Gly supplementation induced significant hyperhydration and substantially attenuated the increase in HR at the end of the 30 min run at 35°C (Figure 5). This attenuation of HR during exercise was of similar magnitude to that previous reported by Easton et al. (2007). As free water in the form of sweat is primarily lost from plasma and since no differences were found in PV changes pre- and post-supplementation despite the increase in TBW, ICW and ECW, it can be suggested that the increase in other water compartments resulted in water moving towards the plasma due to an osmotic gradient. This in turn leaves the PV unaffected. It should be also noted that in order for blood volume to be maintained in conditions of significant thermal stain and therefore sweating, fluid loss is obtained in varying proportions from ECW as well as ICW body water compartments [37]. Furthermore, as loss of body water increases during exercise in the heat as a result of sweating, T_core _also increases [37]. Therefore, increasing body water could potentially result in better maintenance of T_core _during exercise in the heat. Nose et al. [38] reported a strong association between the loss of water in sweat and urine and the decrease in ICW after prolonged exercise in the heat. In the present study, Cr and Gly induced an increase in ICW and consequently, there was a significant attenuation in the rise of T_core _during exercise in the heat (Figure 6). It is possible that this Cr- and Gly-induced increase in ICW resulted in an increase of the specific heat capacity of the body [13].

Published studies to date appear to confirm the reduction of T_core _during exercise in the heat following Cr supplementation [12,13,19]. Conversely, when Gly was used alone, ICW was increased without significantly attenuating the rise in T_core _during the exercise period [19]. The effects of Gly ingestion on T_core _and thermoregulation in general during exercise in the heat is equivocal, with several studies reporting a reduction in T_core _during exercise [39] and numerous other studies finding no such effect [16,40]. In addition, several studies concluded that PV expansion has no effect on thermoregulatory responses or exercise performance during exercise in the heat [9,41]. These conflicting results and assertions provide strong support that the thermoregulatory benefits exhibited with Gly ingestion in the present study did not arise from any PV expansion but most likely from an increase heat capacity of the body. Nevertheless, it should also be noted that these thermoregulatory benefits were exerted when Gly was co-ingested with Cr.

Despite the significant increase in TBW and consequently improvement in cardiovascular and thermoregulatory responses during exercise, no differences in $\dot{\text{V}}{\text{O}}_{\text{2}}$ were observed during running at 60% $\dot{\text{V}}{\text{O}}_{\text{2}}\max$. Coyle proposed that a reduction in BM induced by dehydration would impact on RE during marathon running by reducing the oxygen cost of running [3]. In contrast, hyperhydration should theoretically increase the oxygen cost of running and therefore RE. However, no such effect was found in the present study. Furthermore, there was no increase in $\dot{\text{V}}{\text{O}}_{\text{2}}$ over time during the trial at 10°C. The latter finding indicates that the subjects were working steadily at the calculated individual running speed corresponding 60% of $\dot{\text{V}}{\text{O}}_{\text{2}}\max$. It should be noted that this relatively low intensity was chosen in order to ensure that the present data would be comparable with previous studies conducted under similar conditions [12]. Furthermore, the relatively low intensity was chosen as to secure that all subjects could complete the experiment in the heat while it was high enough to observe possible adaptations in cardiopulmonary or thermoregulatory parameters encountered with supplementation. However, $\dot{\text{V}}{\text{O}}_{\text{2}}$ was increased during the trial in the heat. This was an expected effect as when exercising in hot environmental conditions, T_core _rises accordingly. It has been shown that with an increase in T_core_, $\dot{\text{V}}{\text{O}}_{\text{2}}$ (and therefore RE) also increases [42]. Despite this observation, no discernable difference in $\dot{\text{V}}{\text{O}}_{\text{2}}$ between pre- and post-supplementation trials was reported. No other changes in any of the respiratory variables could be observed in the pre- and post-supplementation trials. Similar results have been reported in several other studies using Cr as the hyperhydrating agent [13] as well as during constant load exercise in the study by Easton et al. (2007) where hyperhydration was induced by Cr and Gly [19]. The data from the present study suggest that an increase in BM of approximately 1.4% (average increase in BM in the present study) has no significant effect on $\dot{\text{V}}{\text{O}}_{\text{2}}$. Whether such an increase in BM would influence running performance remains to be determined. Furthermore, as HR responses reflect those of $\dot{\text{V}}{\text{O}}_{\text{2}}$[43], the finding that HR during exercise was not significantly different between pre- and post-supplementation trials conducted at 10°C is further evidence against any detrimental metabolic effect of the added BM induced by hyperhydration on RE.

## Conclusions

A hyperhydration strategy that combines Cr and Gly supplementation for 7 days increased BM and TBW and consequently reduced cardiovascular and thermal strain but did not significantly affect the oxygen cost of running at 60% of $\dot{\text{V}}{\text{O}}_{\text{2}}\max$ at 35°C in trained runners. The finding that a significant increase in BM did not negatively impact on RE of trained runners, supports the use of effective hyperhydration strategies during endurance running when conditions so dictate (i.e., running in hot and humid conditions). Further studies are necessary however to confirm these findings during faster running speeds reflective of true performance.

## Competing interests

The authors declare that they have no competing interests.

## Authors' contributions

LYB was the primary author of the manuscript. TP was involved in subject recruitment, data collection and helped to draft the manuscript. DM was involved in data collection and editing the manuscript. YPP conceived of the study, participated in its design and coordination and helped to draft the manuscript. All authors read and approved the final manuscript.
